# Different signatures of miR-16, miR-30b and miR-93 in exosomes from breast cancer and DCIS patients

**DOI:** 10.1038/s41598-018-31108-y

**Published:** 2018-08-28

**Authors:** Qingtao Ni, Ines Stevic, Chi Pan, Volkmar Müller, Leticia Oliveira-Ferrer, Klaus Pantel, Heidi Schwarzenbach

**Affiliations:** 1 Department of Tumor Biology, University Medical Center Hamburg-Eppendorf, Hamburg, 20246 Germany; 2 Department of Gynecology, University Medical Center Hamburg-Eppendorf, Hamburg, 20246 Germany

**Keywords:** Breast cancer, Tumour biomarkers

## Abstract

Loading of microRNAs (miRNAs) into exosomes that are involved in cellular communication is a selective process. The current study investigates whether the enrichment of miRNAs in exosomes reflects the pathogenesis of breast cancer (BC) and ductal carcinoma *in situ* (DCIS). The levels of miRNAs were quantified in exosomes from plasma of 32 BC patients, 8 DCIS patients and 8 healthy women by TaqMan real-time PCR-based miRNA array cards containing 47 different miRNAs. Then, exosomal miR-16, miR-30b and miR-93 that displayed deregulation in the arrays were selected and analyzed in 111 BC patients, 42 DCIS patients and 39 healthy women by TaqMan real-time PCR. Identification of exosomes was performed by Western blot. The levels of exosomal miR-16 were higher in plasma of BC (p = 0.034) and DCIS (p = 0.047) patients than healthy women, and were associated with estrogen (p = 0.004) and progesterone (p = 0.008) receptor status. Particularly, in estrogen-positive patients miR-16 was significantly enriched in exosomes (p = 0.0001). Lower levels of exosomal miR-30b were associated with recurrence (p = 0.034). Exosomal miR-93 was upregulated in DCIS patients (p = 0.001). Our findings suggest that different signatures of miR-16, miR-30b and miR-93 in exosomes from BC and DCIS patients are associated with a particular biology of breast tumors.

## Introduction

Breast cancer (BC) is the most frequent cancer type and leading cause of cancer death in women. Up to 10% of women diagnosed with BC develop loco-regional recurrences and up to 30% distant metastases^1^. Gene expression profiling based on the expression of specific receptors, such as estrogen receptor (ER), progesterone receptor (PR) and human epithelial growth factor receptor-2 (HER2), classifies BC into 4 distinct subtypes: luminal A, luminal B, HER2-positive and basal-like (triple negative) BC. Knowledge of the subtype and accordingly the receptor status are required to decide the need of an endocrine therapy and chemotherapy for a BC patient^2^. Ductal carcinoma *in situ* (DCIS) is a potential precursor for invasive BC and comprises approximately 20% of all BC diagnoses^3^. Like BC, DCIS is a heterogeneous disease, and varies in hormonal status, growth factor receptor status, proliferation rate and genetic features. For clinicians the decision between high- and low-risk DCIS is challenging, since a substantial proportion of DCIS probably never becomes invasive^4^.

In general, tumors are associated with different genetic and epigenetic events resulting in a change of gene expression. Alterations, like the deregulation of microRNAs (miRNAs) can be detected also in different liquid biopsies. Currently, the selective loading of miRNAs into exosomes that circulate in high amounts in the bloodstream of cancer patients is of growing interest^5,6^. MiRNAs are a family of evolutionary conserved, small non-coding RNA molecules consisting of approximately 22 nucleotides. They inhibit gene expression post-transcriptionally by binding specifically to the 3′ untranslated-region (3′UTR) of their target mRNAs. Gene silencing occurs through translational inhibition or cleavage of their target mRNAs^7^. Due to their binding affinity to hundreds of different mRNAs, miRNAs regulate numerous signal transduction pathways. Among others they participate in development, differentiation, proliferation, tumor development and progression^8^. In mammals, they are assumed to regulate approximately 50% of all protein-coding genes^9^. MiRNAs are actively secreted into the blood circulation by exosomes which belong to microvesicles^10^. Their involvement in cell-to-cell communication provokes exosomes to propagate cancer by transferring their cargo from cancer to healthy cells^11,12^. For example, miRNAs discharged in the recipient cells by exosomes can be functional there and alter the characteristics of these cells. Respective *in vitro* and *in vivo* observations corroborate the association of an increased secretion of exosomes with tumor invasiveness and metastasis^13^. We also detected high concentrations of exosomes in the plasma/serum of BC and epithelial ovarian cancer patients, as well as higher concentrations of exosomal miRNAs than cell-free miRNAs^14,15^.

In the present study, we found that deregulated levels of exosomal miR-16, miR-30b and miR-93 were most significantly associated with BC receptor status, BC recurrence and DCIS, respectively.

## Results

### Workflow

**Figure 1:**
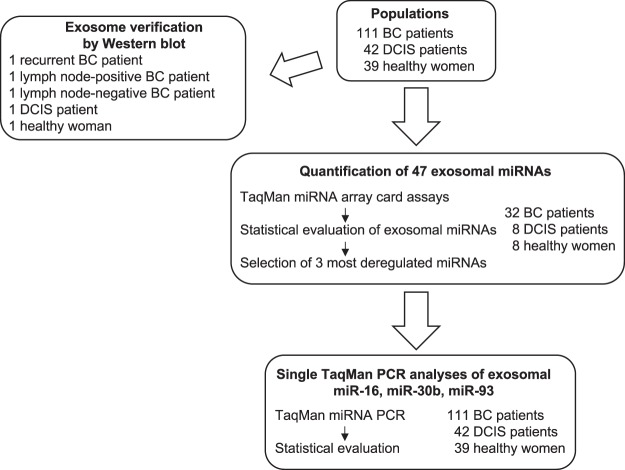
Workflow of the present study.

At first, real-time PCR-based miRNA array cards containing 45 miRNAs (plus 2 references and a blank control) were used to quantify miRNAs in exosomes derived from plasma samples of 32 BC patients, 8 DCIS patients and 8 healthy women. Then, three significantly deregulated exosomal miRNAs (miR-16, miR-30b and miR-93) derived from these array card analyses were selected for single TaqMan real-time PCR assays using exosomes from plasma of 111 BC patients, 42 DCIS patients and 39 healthy women. The relative miRNA data normalized by the endogenous miR-484 and exogenous cel-miR-39 were statistically evaluated, and compared among the cohorts and with the clinical parameters of the BC patients. Exosomes were verified in 3 BC patients, 1 DCIS patient and 1 healthy woman by a Western blot. Figure 1 summarizes the single steps of the workflow.

### Verification of exosomes

**Figure 2:**
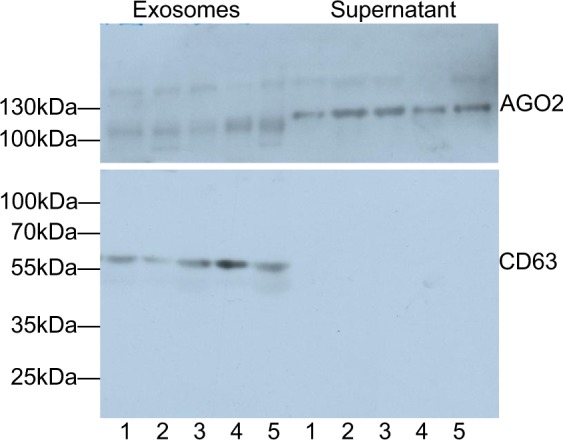
Verification and quantification of exosomes. Exosomes were precipitated from plasma of a healthy woman, DCIS patient and BC patients by the agglutinating agent ExoQuick and analyzed by Western blots using antibodies specific for the exosomal marker CD63, and the miRNA-associated AGO2 protein. The Western blots show representative examples of exosomes, devoid of cell-free miRNAs. Lane 1, healthy woman; lane 2, DCIS patient; lane 3, lymph node-negative BC patient; lane 4, lymph node-positive BC patient; lane 5, recurrent BC patient.

Prior to quantification of exosomal miRNAs, the extractions of exosomes from a healthy woman and a patient with DCIS, lymph node-negative BC, lymph node-positive BC and recurrent BC were verified on a Western Blot using antibodies specific for the exosomal marker CD63 and the miRNA binding protein AGO2. As shown by the bands around the 55 kDa and between 100 and 130 kDa on the blot, the CD63-specific antibody recognized non-lysed exosomes in the pellet, whereas the AGO2-specific antibody did not detect AGO2 protein which is bound to cell-free miRNAs in the exosome pellet, respectively. Conversely, exosome- and cell-free AGO2-bound miRNAs could only be found in the supernatant (Fig. 2). These findings show that the exosome fraction may be pure and devoid of cell-free miRNAs. However, they do not exclude that the exosomes may contain traces of contaminations of AGO2-bound miRNAs that due to the sensitivity of the Western blot were not detectable (Fig. 2).

### MiRNA profiling in exosomes of BC and DCIS patients

**Figure 3:**
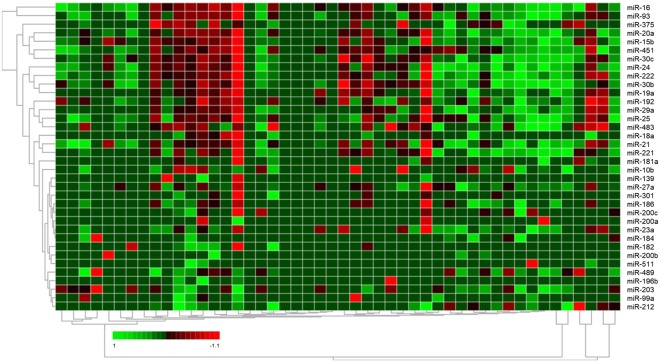
Hierarchical cluster of 47 exosomal miRNAs. The heat map is derived from data of the miRNA array assays which were carried out by quantitative real-time PCR-based array cards mounted with assays for detection of 47 different miRNAs and using exosome samples from plasma of 32 BC patients, 8 DCIS patients and 8 healthy women. The colored representation of samples and probes is ordered by their similarity. The red and green colors indicate that the ΔCq value is below (relatively high expression) and above (relatively low expression levels) the median of all ΔCq values in the study, respectively. On top: clustering of samples. On the right side: clustering of probes. The scale bar provides information on the degree of regulation.

**Figure 4:**
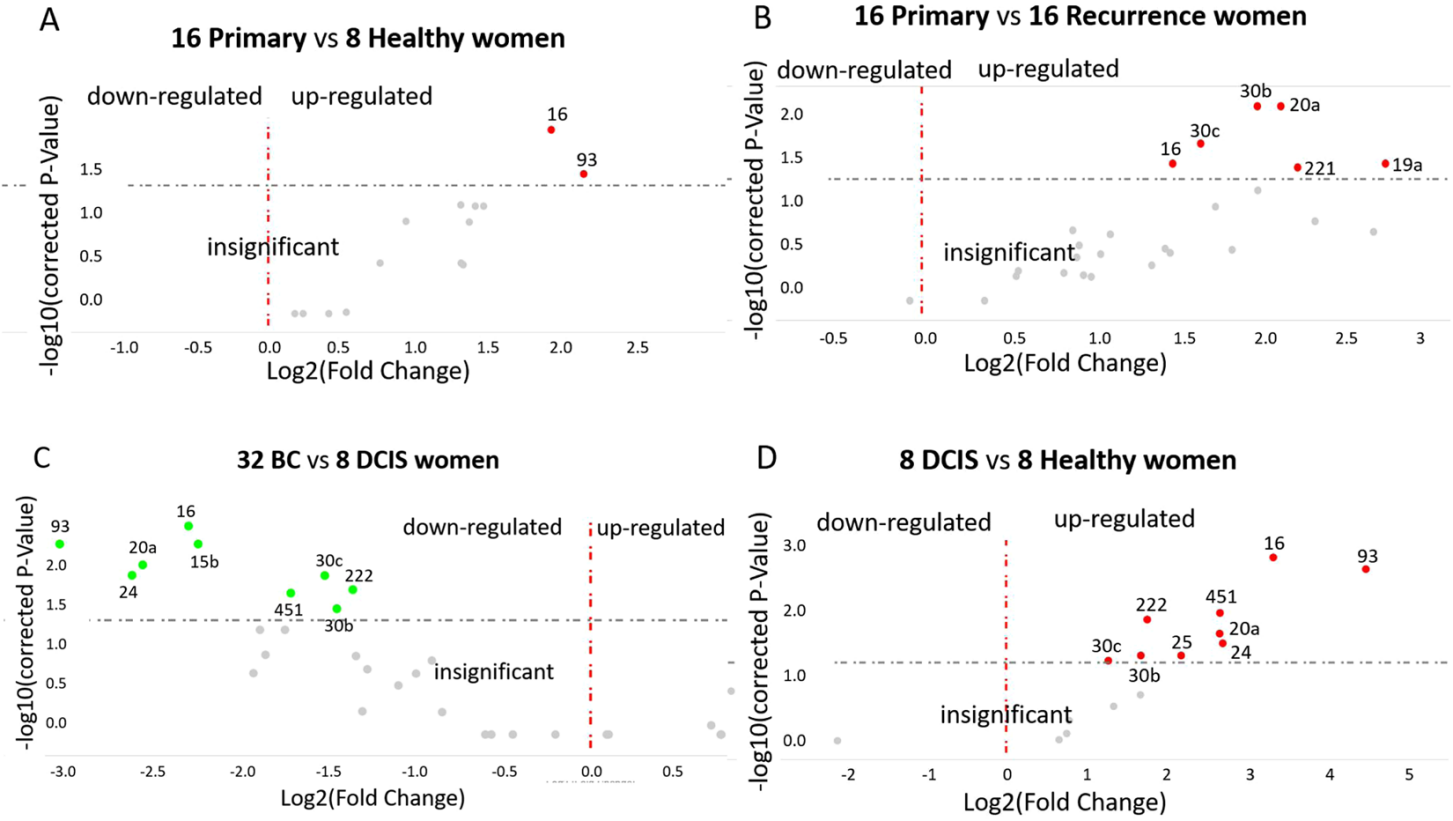
Volcano plots of exosomal miRNAs. The plots were drawn for comparison of exosomal miRNA levels in plasma of 16 primary BC patients with those of 8 healthy women (**A**) and 16 primary BC patients with recurrence (**B**) as well as of 32 BC patients with 8 DCIS patients (**C**), and of 8 DCIS patients with 8 healthy women (**D**). The Log2 fold changes are plotted on the x-axis and the negative log10 p-values are plotted on the y-axis. The left side shows downregulated exosomal miRNAs (green dots). The right side shows upregulated exosomal miRNAs (red dots). Under the dashed horizontal line there are non-deregulated miRNAs (grey dots).

Next, we carried out a quantitative TaqMan real-time PCR-based microarray with cards containing 45 different miRNAs (plus 2 references and a blank), to determine the miRNA expression profiles in exosomes derived from the plasma of 32 BC (16 primary and 16 recurrent) patients, 8 DCIS patients and 8 healthy women (Fig. 1). The selected miRNAs are listed in Materials and Methods. We selected these miRNAs for the assembly of the 48-miRNA array cards due to our previous studies^14,16–18^. Briefly, in our previous studies, we found a prevalence of miR-101, miR-372 and miR-373 in exosomes^14^, and significantly higher plasma levels of cell-free miR-16 and miR-27a^16^, as well as higher serum levels of cell-free miR-21 and miR-210 in BC patients than in healthy women. Moreover, we observed an association of miR-21 levels before and after chemotherapy with overall survival of BC patients^17^. Finally, we found that serum concentrations of deregulated miR-93, miR-155 and miR-373 may be linked to a particular biology of BC favoring progression and metastatic spread^18^. From the literature on PubMed, we selected the other miRNAs for the array cards. These miRNAs were shown to play a role in tumorigenesis and progression, and were involved in regulation of the cell cycle, apoptosis, proliferation, migration and invasion^19,20^. Derived from the data of this real-time PCR-based miRNA array, a similarity matrix was generated containing all pairwise similarities of the exosome samples from plasma of BC patients, DCIS patients and healthy controls. To detect potential clusters in rows (miRNAs) and columns (plasma samples) of the normalized expression matrix, hierarchical clustering was carried out. The relative up- and downregulated miRNAs are indicated by red and green, respectively (heat map, Fig. 3). As shown in the Volcano plot, exosomal miR-16 (p = 0.014) and miR-93 (p = 0.038) were upregulated in primary BC patients compared with healthy women (Fig. 4A). The levels of 6 exosomal miRNAs differed between primary and recurrent BC. In particular, the levels of miR-20a (p = 0.008) and miR-30b (p = 0.008) were higher in exosomes from primary than recurrent BC patients (Fig. 4B). In each case, the levels of 9 miRNAs could distinguish DCIS from BC patients (Fig. 4C) and healthy women (Fig. 4D). We selected exosomal miR-16, miR-30b and miR-93 for single TaqMan PCR assays, because the increased levels of miR-16 in DCIS patients compared with healthy women (p = 0.001) decreased in all BC patients (p = 0.004) and recurrent patient (p = 0.001). Alike, the levels of miR-30b were higher in DCIS patients than healthy women (p = 0.038) and decreased in all BC patients (p = 0.037) and recurrent BC patients (p = 0.003). The levels of miR-93 were also higher in DCIS patients than healthy women (p = 0.001), but decreased in all BC patients (p = 0.007) and recurrent BC patients (p = 0.002, Fig. 4, Supplementary Table S1).

Then, we analyzed the selected miR-16, miR-30b and miR-93 in exosomes from plasma of 111 BC patients, 42 DCIS patients and 39 healthy women by single TaqMan real-time PCR assays (data summarized in Supplementary Table S2). The higher levels of exosomal miR-16 in all BC patients than in healthy women (p = 0.034) were not observed in primary BC patients, but in recurrent patients (p = 0.016), indicating that this increase was possibly linked with recurrence. The deregulated levels of miR-16 in DCIS patients compared with those of healthy women and all BC patients were borderline (p = 0.047). No changes in the levels of exosomal miR-30b could be observed in primary and recurrent BC patients, as well as in DCIS patients (Table 1). These findings were different from the data derived from the analyses using the array cards, suggesting that the number of patients and healthy women was too low in the array analyses to get robust p-values. However, the significant increase in the levels of exosomal miR-93 in DCIS patients using array cards could also be found in these patients by single PCR assays (p = 0.001, Table 1, Supplementary Fig. S2).

**Table 1:** Different enrichments of miR-16, miR-30b and miR-93 in exosomes.

Populations	No.			miR-16	miR-30b	miR-93
All BC vs. Healthy	111 vs. 39		fold change	**2.1**	0.9	**1.2**
			p-value	**0.034**	0.903	**0.046**
Primary vs. Healthy	65 vs. 39		fold change	1.9	1.1	1.4
			p-value	0.067	0.538	0.057
Recurrence vs. Healthy	46 vs. 39		fold change	**2.5**	0.7	1.1
			p-value	**0.016**	0.195	0.345
Recurrence vs. Primary	46 vs. 65		fold change	1.3	**0.7**	0.8
			p-value	0.214	**0.034**	0.151
DCIS vs. Healthy	42 vs. 39		fold change	**1.8**	0.9	**2.0**
			p-value	**0.047**	0.870	**0.001**
All BC vs. DCIS	111 vs. 42		fold change	**1.2**	1.0	**0.6**
			p-value	**0.047**	0.732	**0.046**
Primary vs. DCIS	65 vs. 42		fold change	1.1	1.2	0.7
			p-value	0.326	0.707	0.465
Recurrence vs. DCIS	46 vs. 42		fold change	**1.4**	0.8	**0.5**
			p-value	**0.029**	0.224	**0.005**
**BC subtypes**						
Histology	21 vs. 77		fold change	1.1	**0.7**	1.3
lobular/tubular vs. other types			p-value	0.785	**0.024**	0.300
ER status	81 vs. 28		fold change	**1.5**	1.1	1.5
positive vs. negative			p-value	**0.004**	0.537	0.201
PR status	74 vs. 35		fold change	**1.4**	1.2	**1.9**
positive vs. negative			p-value	**0.008**	0.953	**0.010**
Triple-negative	24 vs. 85		fold change	**0.7**	0.8	0.7
yes vs. no			p-value	**0.012**	0.891	0.296

P-values with the corresponding fold changes of exosomal miRNAs in bold.

### Association of exosomal miR-16, miR-30b and miR-93 with clinicopathological parameters of BC patients

The statistical comparison with clinical data shows that the levels of exosomal miR-16 are associated with the receptor status of BC patients. The levels were higher in ER-positive BC patients than in healthy women (p = 0.0001, Fig. S2). Exosomal miR-16 could differ between ER-positive and -negative (p = 0.004), as well as between PR-positive and -negative (p = 0.008) BC patients. Triple-negative patients had lower level of miR-16 than the receptor-positive patients (p = 0.012, Table 1). These findings show that the increased levels of miR-16 in exosomes from recurrent BC patients decreased in receptor-negative patients. Moreover, the levels of exosomal miR-30b and miR-93 could distinguish lobular/tubular BC from other histological subtypes (p = 0.024) and PR-positive from -negative BC (p = 0.010), respectively (Table 1).

## Discussion

In the present study, we analyzed miR-16, miR-30b and miR-93 signatures in exosomes derived from plasma of BC patients and compared them with those in DCIS patients and healthy women using quantitative TaqMan real-time PCR-based miRNA array cards and single PCR assays. At first, we verified the extracted exosomes by immunoblotting using an antibody specific for the exosomal marker CD63, and detected a CD63 band around 55 kDa on the blot, indicating that the antibody recognized non-lysed exosomes in the pellet. Our proceeding studies whose data will be published in a future study show that the majority of these exosomes are of 120 nm in diameter using Nanoparticle Tracking Analysis on a Nanosight device. In the literature, the molecular weight (MW) of exosome-derived CD63 has been described to be around 50 kDa^21,22^, a bit lower than our measured MW of 55 kDa, but Xu *et al*. observed that the MW range of CD63 was from 30 to 75 kDa under non-reducing conditions^23^. Tominaga *et al*. characterized the MW of CD63 more precisely. They found MWs of 25 kDa for non-glycosylated, 35 kDa for lower-glycosylated and above 50 kDa for higher-glycosylated CD63^24^. However, all these studies measured the exosomes in cell supernatants. We only found two articles which characterized exosomes in plasma and serum. Surprisingly, Poon *et al*. detected exactly the exosomal protein band of CD63 at MW of 63 kDa on a Western blot using plasma from patients with cardiopulmonary bypass^25^, while Kim *et al*. detected a broad band around 50 kDa using pooled normal human serum samples^26^. This broad range of MWs of CD63 detected in the diverse studies can be explained by the use of different sources (cells, plasma or serum), technical platforms (polymeric solutions or ultracentrifugation) and the glycosylation of CD63 protein.

Then, we compared the miRNAs data derived from quantitative TaqMan real-time PCR-based miRNA array cards with those of single PCR assays. We found that the data derived from these both techniques were only partly overlapping, suggesting that the numbers of patients and healthy women were too low in the array assays to get robust p-values. It is rather unlikely that the difference in data is caused by the use of two different techniques because the tendency of the deregulation of these three miRNAs is similar. In particular, the significant enrichment of miR-93 in exosomes from DCIS patients compared with healthy women and BC patients was detected by both techniques. Although our patient cohorts are small and further analyses are necessary, our findings point to an association of exosomal miR-93 with DCIS. Approximately one of five breast tumors detected by mammography is diagnosed as DCIS^4^, but it is still challenging to distinguish DCIS cases that are likely to progress from cases that remain indolent, and therefore do not require intensive treatment. In this regard, Lesurf *et al*. established a specific signature able to identify pre-invasive DCIS harboring a miRNA expression profile that resembles invasive carcinoma, indicating that these DCIS cases have a higher likelihood of future disease progression^27^. Concerning miR-93, further follow-up analyses are necessary to determine whether the enrichment of this miRNA in exosomes also reflects a possible future invasiveness of DCIS. Particularly, considering the high cancer-specific secretion of exosomes that are important mediators between tumor and its microenvironment, the exosome shuttle of miR-93 could contribute to increase cell invasion^28^. By the way, it was reported that upregulation of miR-93 promoted cell migration, invasion and proliferation in BC, as well as participated in regulating angiogenesis in cancers^29–31^. Moreover, PTEN seems to be a target of miR-93, and consequently, miR-93 may regulate the activity of PI3K/Akt pathway^29^. Previously, we observed higher transcript concentrations of circulating miR-93 in primary BC patients than in healthy women, but not in BC patient with metastasis^18^. However, in the present study the enrichment of miR-93 in exosomes from BC patients was only of borderline significance. To date, we only found one article that quantified miR-93 in exosomes from BC patients. This study by Sueta *et al*. showed that the levels of miR-93 in tumor tissues were upregulated, but downregulated in exosomes from serum of patients with recurrence compared with those with no recurrence^32^. We did not observe such an association of exosomal miR-93 with recurrence. This discrepancy could be explained by the small cohort used in this study for the quantification of exosomal miR-93, namely 16 recurrent and 16 non-recurrent BC patients. Nonetheless, these data demonstrate differently deregulated levels of miRNAs between exosomes and tumor tissues, and a selective packaging process of miRNAs into exosomes that is not related to their expression levels in the primary tumor.

In our study, miR-16 was differentially enriched in exosomes of BC patients compared with healthy women, making it an excellent biomarker candidate for BC diagnosis. However, miR-16 is also deregulated in other cancer types^33^, influencing the accuracy of BC diagnosis. A clinically applicable miRNA as independent biomarker for a specific cancer type has not yet been identified. Moreover, at the systemic level, a single biomarker is unlikely to conduct the complicated process of cancer development. Therefore, a panel of specific miRNAs or the combination of such a miRNA, with other conventional clinical biomarkers or physical and pathological examinations might improve the diagnostic power. In addition, sensitivity and specificity are the two major standards for an accurate diagnosis tool. Based on the frequent heterogeneous miRNA expression across BC patients, an appropriate standard cut-off value for miR-16 levels is required for the determination of BC diagnosis. However, discrepant data on miR-16 exist in numerous publications that describe this miRNA as tumor suppressor gene, oncogene or reference miRNA^19,20,34^. Our findings show that the enrichment of miR-16 in exosomes is especially associated with recurrence and receptor status. We found higher levels of exosomal miR-16 in ER- and PR-positive BC patients than in ER-, PR- and triple negative BC patients, as well as higher levels in BC patients with recurrence than in healthy women and DCIS patients. To sum up, our data suggest a selective and wavelike packaging of miR-16 into exosomes in the different BC subtypes, and possibly during tumor development and progression. As far as we know, there is no study quantifying miR-16 in exosomes from BC patients. To date, our previous data showed that the increased plasma levels of miR-16 in BC patients compared with healthy women were associated with lymph node status, but they did not provide any indication of an association with the receptor status^16^, suggesting, again that higher levels of miR-16 in plasma do not implicate a higher enrichment in exosomes. Interestingly, Usmani *et al*. reported higher levels of miR-16 in serum of invasive intraductal BC patients and their daughters than in healthy women, and described, therefore, miR-16 as an hereditary marker in BC^35^. Beside BC, upregulated miR-16 levels in tissue of renal cell cancer (RCC) patients were also found. In RCC cell lines, miR-16 acted as an oncogene by inducing cellular proliferation, migration and reducing apoptosis^36^. These findings suggest that miR-16 is involved in numerous biological processes in different tumor types, and may have several different mRNA targets. For example, Gattolliat *et al*. showed that in colorectal cancer (CRC) tissue the decreased expression of anti-apoptotic protein BCL2, a potential target of miR-16, was possibly caused by the upregulation of miR-16^37^. In contrast, Ma *et al*. showed that miR-16 was downregulated in majority of CRC tissues, and overexpression of miR-16 inhibited proliferation and induced apoptosis of CRC cells. They revealed that miR-16 repressed CRC cell growth *in vitro* by regulating the p53/survivin signaling pathway. In this process, miR-16 repressed the expression of its mRNA target survivin and reduced p53 expression. Conversely, p53 increased miR-16 levels, with downregulation of miR-16 targets survivin, cyclin D1 and CDK6^38^. In BC cells, miR-16 downregulated the oncogenes BCL2 and BMI1 (B lymphoma Mo-MLV insertion region 1), that regulates the chromatin structure and is involved in the self-renewal of stem cells, but upregulated pro-apoptotic proteins and mitochondrial reactive oxygen species. This led to intrinsic apoptosis mediated by impaired mitochondrial membrane potential, followed by cytochrome-C release into the cytosol that activated Caspase-3 and Caspase-6/9^39^. Furthermore, the down-regulation of BMI1, which also impedes DNA repair, by miR-16 could sensitize BC cells to doxorubicin resulting in apoptotic cell death^40^. Finally, miR-16 was described as a potential therapeutic target and clinical biomarker of bone metastasis. Elevated levels of miR-16 were detected in mice with different bone metastatic tumor burdens, and also in the serum of BC patients with bone metastases, reflecting the presence of osteoclasts. The detection of increased levels of miR-16 along with those of soluble intracellular adhesion molecule which is involved in the activation of NFκB signaling in bone lesions shows the potential for their use as potential targets in the treatment of aberrant osteoclast activity^41^. To sum up, these investigations reveal the multiple behaviors of miR-16, and accordingly, the shortcomings of a replacement therapy with miR-16. Therefore, in a temporal and local context, the up- and downregulation of miR-16 need to be taken into contextual evaluation, whether its delivery or targeting may be beneficial in BC^42^. In this regard, the diverse enrichments of miR-16 in exosomes derived from the different BC subtypes, as detected in our study, along with the exosome shuttle have to be further analyzed. A hypothesis could be that these exosomes transfer miR-16 to other cells to induce apoptosis via BCL2, but it should be kept in mind that exosomes also transfer other miRNAs.

In our study, we detected no deregulated levels of miR-30b in exosomes from BC patients, but the levels of exosomal miR-30b could differ between primary and recurrent BC, as well as between lobular/tubular and other histological BC. Interestingly, a study by Tormo *et al*. showed that miR-30b may regulate trastuzumab resistance and Cyclin E2 gene in HER2-positive BC patients^43^.

In conclusion, our findings show a specific packaging of miR-16, miR-30b and miR-93 into exosomes from BC and DCIS patients. In particular, miR-93 was significantly enriched in exosomes from DCIS patients. Further follow-up studies are needed to investigate whether exosomal secretion of miR-93 may impact signaling in nearby breast cells and stimulate their invasiveness.

## Methods

### Study populations and plasma samples

Plasma samples were collected from 111 BC and 42 DCIS patients treated at the University Medical Center Hamburg-Eppendorf, Department of Gynecology. In addition, plasma samples were collected from 39 healthy women who had no history of cancer and were in good health based on self-report. Median age of BC patients, DCIS patients and healthy women was 63 (27 to 92), 58 (30 to 76) and 59 (49 to 71) years, respectively. Plasma samples of BC patients collected directly before surgery and DCIS patients were obtained from November 2013 to September 2016. Those of healthy women were obtained from September 2015 to January 2018, respectively. All patients gave written informed consent to access their blood and review their medical records according to our investigational review board and ethics committee guidelines. Blood collection and experiments were performed in compliance with the Helsinki Declaration and were approved by the ethics committee (Ethik-Kommission der Ärztekammer Hamburg, Hamburg, PV5392). Regarding blood processing, uniform management concerning the specific, described protocols was performed. Clinicopathological parameters details are described in Table 2.

**Table 2:** Clinicopathological parameters of breast cancer patients.

Breast cancer patients	111
age	63 (27–92)
Recurrence	
yes	46 (41.4%)
no	65 (58.6%)
CT stage	
CT 1	47 (42.3%)
CT 2–3	60 (54.1%)
unknown	4 (3.6%)
Grading	
G 1–2	60 (54.1%)
G 3	45 (40.5%)
unknown	6 (5.4%)
Histology	
lobular/tubular	21 (18.9%)
other types	77 (69.4%)
unknown	13 (11.7%)
Nodal status	
negative	74 (66.7%)
positive	31 (27.9%)
unknown	6 (5.4%)
Lymph-invasion	
0	74 (66.7%)
1	27 (24.3%)
unknown	10 (9%)
ER status	
negative	28 (25.2%)
positive	81 (73.0%)
unknown	2 (1.8%)
PR status	
negative	35 (31.5%)
positive	74 (66.7%)
unknown	2 (1.8%)
Triple-negative	
yes	24 (21.6%)
no	85 (76.6%)
unknown	2 (1.8%)

### Verification of hemolysis in plasma samples

To avoid analyzing hemolytic plasma samples that may influence the concentrations of exosomal miRNAs, we carried out hemoglobin measurements by spectral analysis. Red blood cells were lysed in 7 ml whole blood by erythrocyte lysis buffer (containing 0.3 M sucrose, 10 mM Tris pH 7.5, 5 mM MgCl_2_ and 1% Triton X100). A dilution series (1:1, 1:3, 1:4, 1:6, 1:8, 1:10, 1:12, 1:14, 1:18, 1:20) of lysed red blood cells was prepared in plasma and served as a standard curve for the measurement of hemolysis in all plasma samples. Fifty µl of standard and plasma samples to be analyzed were measured in duplicates on a Microplate reader (Tecan, Männerdorf, Switzerland). Absorbance peaks at 414, 541 and 576 nm were indicative for free hemoglobin, with the highest peak at 414 nm. The higher the absorbance in plasma samples is the higher the degree of hemolysis. The average values were calculated from the duplicates (Supplementary Fig. S1).

### Exosome extraction

Exosomes were isolated from plasma samples with the ExoQuick kit (BioCat, Heidelberg, Germany). Plasma was centrifuged at 3,000 g for 15 min. to remove cell debris. Then, 120 µl ExoQuick Exosome Precipitation Solution were added to 500 µl plasma. The plasma-ExoQuick mixture was incubated at 4 °C for 30 min, and subsequently, centrifuged at 1,500 g, for 30 min, and at 1,500 g, for 5 min. The pellet contained the exosomes.

### Western blot

The protein concentrations were measured with the DC Protein Assay Kit (BioRad, Munich, Germany) at a wavelength of 650 nm on a spectrophotometric plate reader (Tecan). A standard curve of 0, 0.15625, 0.3125, 0.625, 1.25, 2.5, 5 and 10 mg/ml BSA (bovine serum albumin; Sigma Aldrich Chemie, Munich, Germany) was applied. Then, 2.5 μl of exosomes and BSA standard samples, resuspended in PBS (Phosphate-Buffered Saline) buffer (Life Technologies, Paisley, UK) were added to 96-well plates. For Western blot, 30 μg of exosomes resuspended in PBS buffer (Life Technologies) were electrophoretically separated and blotted onto a PVDF (polyvinylidene difluoride) membrane (Millipore, Billerica, USA) which was subsequently incubated with antibodies specific for the exosomal marker CD63 (Abgent, San Diego, California, USA) and the miRNA binding protein AGO2 (Takara Bio Inc., Shiga, Japan) overnight. Detection of the proteins was carried out using a peroxidase-conjugated secondary antibody (Dako, Glostrup, Denmark) and a chemiluminescence ECL detection solution (Sigma-Aldrich St.Louis, Missouri, USA). Images were acquired by using Microsoft Paint system (Microsoft, Washington, USA).

### Extraction of miRNAs from exosomes

MiRNAs were extracted from exosomes resuspended in 150 µl lysis buffer and 50 µl PBS by using the TaqMan miRNA ABC Purification Buffer Kit (Thermo Fisher Scientific, Vilnius, Lithuania). According to the manufacturer’s instructions, the exosomal miRNAs were bound to 80 µl anti-miR beads using the TaqMan miRNA ABC Purification Bead kit Human panel A (Thermo Fisher Scientific). To avoid technical variability, 2 µl of 1 nM synthetic non-human cel-miR-39 were added as an exogenous spike in control.

### Conversion of exosomal miRNAs into cDNA

Reverse transcription was carried out using modified protocols of TaqMan MicroRNA Reverse Transcription kit (Thermo Fisher Scientific). For PCR-based TaqMan miRNA array, the reaction contained 6.0 µl Custom RT Primer Pool, 0.3 µl dNTPs with 100 mM dTTP, 3.0 µl (50 U/μl) MultiScribe Reverse Transcriptase, 1.5 µl 10xRT Buffer, 0.19 µl (20 U/μl) RNase Inhibitor and 4 µl exosomal miRNAs. For single TaqMan PCR analyses, the reaction contained 4.0 µl RT Primer Pool [RT primer of miR-484, cel-miR-39, miR-16, miR-30b and miR-93 mixture diluted in TE (Tris-EDTA) 1:100], 0.2 µl dNTPs with 100 mM dTTP, 2.0 µl (50 U/μl) MultiScribe Reverse Transcriptase, 1 µl 10xRT Buffer, 0.127 µl (20 U/μl) RNase Inhibitor, and 2 µl exosomal miRNAs. The reactions were carried out at 16 °C for 30 min, 42 °C for 30 min and 85 °C for 5 min on an MJ Research PTC-200 Peltier Thermal Cycler (Global Medical Instrumentation, Ramsey, Minnesota, USA).

### Preamplification of cDNA

To increase input cDNA, a preamplification step of cDNA was included. For PCR-based TaqMan miRNA array analyses, 25 µl preamplification reaction mix contained 12.5 µl TaqMan PreAmp Master Mix, 3.75 µl Custom PreAmp Primer Pool (Thermo Fisher Scientific) and 5 µl cDNA. For single TaqMan PCR analyses of miR-16, miR-30b and miR-93, cDNA of the reference miR-484 and miR-39 was also preamplified. Here, 1 μl cDNA were preamplified in 5 μl Taqman PreAmp Master Mix (Thermo Fisher Scientific) and 1.5 μl PreAmp primer pool (TaqMan miRNA primers of miR-484, cel-miR-39, miR-16, miR-30b and miR-93 mixture diluted in TE 1:100). The reactions were run on a MJ Research PTC-200 Peltier Thermal Cycler (Global Medical Instrumentation): 1 cycle at 95 °C for 10 min, 55 °C for 2 min, 72 °C for 2 min; 16 cycles at 95 °C for 15 s, 60 °C for 4 min; and a final cycle 99.9 °C for 10 min. A negative control without any templates was included from the starting point of reverse transcription, too.

### PCR-based TaqMan miRNA arrays

Custom real-time PCR-based TaqMan miRNA array cards (Thermo Fisher Scientific) were used for miRNA profiling. This array cards contain assays for the detection of 45 human miRNAs of interest, 1 endogenous reference miRNAs (miR-484) and 1 exogenous reference miRNA (cel-miR-39-3p) for data normalization and 1 assay with an N/A-4343438-Blank (negative control). For the array cards, we selected the 45 miRNAs of interest because they have previously been described to be clinically relevant and with an exclusive consideration for BC. These miRNAs of interest were then mounted on the array cards by the company Thermo Fisher Scientific and are as follows: RNU6, miR-1, miR-10b, miR-15b, miR-16, miR-18a, miR-19a, miR-20a, miR-21, miR-23a, miR-24, miR-25, miR-27a, miR-29a, miR-30b, miR-30c, miR-31, miR-34c, miR-93, miR-99a, miR-135b, miR-139-3p, miR-181a, miR-181c, miR-182, miR-184, miR-186, miR-192, miR-196b, miR-200a, miR-200b, miR-200c, miR-203, miR-205, miR-210, miR-212, miR-221, miR-222, miR-301, miR-375, miR-451, miR-483-5p, miR-489, miR-492, miR-511.

For miRNA array analyses, we modified the protocol of Thermo Fisher Scientific as followed: The 112.5 µl PCR reaction contained 56.25 µl TaqMan Universal Master Mix II and 2 µl PreAmp product. PCR array cards were run on a 7900 HT Fast Real-Time PCR System (Applied Biosystems): 1 cycle at 95 °C for 10 min, 40 cycles at 95 °C for 15 sec and 60 °C for 1 min.

### Single TaqMan PCR analyses of miR-16, miR-30b and miR-93

For quantitative real-time PCR, the TaqMan miRNA assays (Thermo Fisher Scientific) for miR-484 and miR-39 (reference miRNAs), and miR-16, miR-30b and miR-93 were used. In a 10 μl-reaction, 0.25 μl preamplified cDNA were mixed with 5 μl TaqMan Universal PCR Master Mix and 0.5 μl TaqMan MicroRNA Assay Quantitative real-time PCR reaction was performed at 95 °C for 10 min. and in 40 cycles at 95 °C for 15 s and 60 °C for 60 s, on a C1000 Touch real-time PCR device (Bio-Rad, Hercules, California, USA).

### Data normalization and statistical analyses

Data analyses were performed using the Thermo Fisher Scientific Analysis Software, Relative Quantification Analysis Module, version 3.1 (www.aps.thermofisher.com), and SPSS software package, version 22.0 (SPSS Inc. Chicago, IL).

We chose miR-484 and cel-miR-39 as an endogenous and exogenous reference gene, respectively, to normalize our miRNA data. MiR-484 showed the smallest variation between healthy individuals, DCIS patients and BC patients. The inter-individual variability of the efficiency of our procedures was controlled by spiking of cel-miR-39-3p. The obtained data of the miRNA expression levels were calculated by the ΔCt method as follows: ΔCt = mean value Ct (reference cel-miR-39 + miR-484) − mean value Ct (miRNA of interest). The Thermo Fisher Scientific Analysis Software was used for performing hierarchical clustering (heat map) and volcano plots. Distances between samples and assays were calculated for hierarchical clustering based on the ΔCt values using Pearson’s Correlation. Clustering method was average linkage. Subsequently, the relative expression data were 2^(ΔCt)^ transformed in order to obtain normal distribution data. The confidence of 2^(ΔCt)^ data were verified by amplification curves and Ct confidence (0-1, whereby 1 refers to the highest confidence). Our data showed a Ct confidence of 0.95. Values below 0.95 were discarded. Statistical differences between populations were calculated using two-tailed student t-test and depicted as volcano plots. The correlations of miRNAs amounts with clinical parameters were calculated by two-tailed student t-test. A p-value < 0.05 was considered as statistically significant.

## Electronic supplementary material

Supplementary information

## Data Availability

All data generated or analyzed during this study are included in this article (and its Supplementary Information files).
