# Author Correction: Different signatures of miR-16, miR-30b and miR-93 in exosomes from breast cancer and DCIS patients

**DOI:** 10.1038/s41598-019-44070-0

**Published:** 2019-12-05

**Authors:** Qingtao Ni, Ines Stevic, Chi Pan, Volkmar Müller, Leticia Oliveira-Ferrer, Klaus Pantel, Heidi Schwarzenbach

**Affiliations:** 1 Department of Tumor Biology, University Medical Center Hamburg-Eppendorf, Hamburg, 20246 Germany; 2 Department of Gynecology, University Medical Center Hamburg-Eppendorf, Hamburg, 20246 Germany

Correction to: *Scientific Reports* 10.1038/s41598-018-31108-y, published online 28 August 2018

The original version of this Article contained a typographical error in the spelling of the author Leticia Oliveira-Ferrer, which was incorrectly given as Leticia Oliviera-Ferrer. This has now been corrected in the PDF and HTML versions of the Article, and in the accompanying Supplementary Information file.

