# Supplementary material for: Different signatures of miR-16, miR-30b and miR-93 in exosomes from breast cancer and DCIS patients
Source: Sci Rep. 2018 Aug 28;8:12974. doi: 10.1038/s41598-018-31108-y (PMC6113263; doi:10.1038/s41598-018-31108-y)
Supplement: Supplementary file 1 — Supplementary information [file 41598_2018_31108_MOESM1_ESM.pdf]

Different signatures of miR-16, miR-30b and miR-93 in exosomes from breast cancer and DCIS patients

Qingtao Ni[1], Ines Stevic[1], Chi Pan[1], Volkmar Müller[2], Leticia Oliveira-Ferrer[2], Klaus Pantel[1], Heidi Schwarzenbach[1]*

[1]Department of Tumor Biology, University Medical Center Hamburg-Eppendorf, Hamburg, 20246, Germany

[2]Department of Gynecology, University Medical Center Hamburg-Eppendorf, Hamburg, 20246, Germany

*Correspondence to:

Heidi Schwarzenbach, PhD

Department of Tumor Biology

University Medical Center Hamburg-Eppendorf

Martinistraße 52

20246 Hamburg, Germany

Phone: +49 40 7410 57494

Fax: +49 40 7410 56546

Email: h.schwarzenbach@uke.de

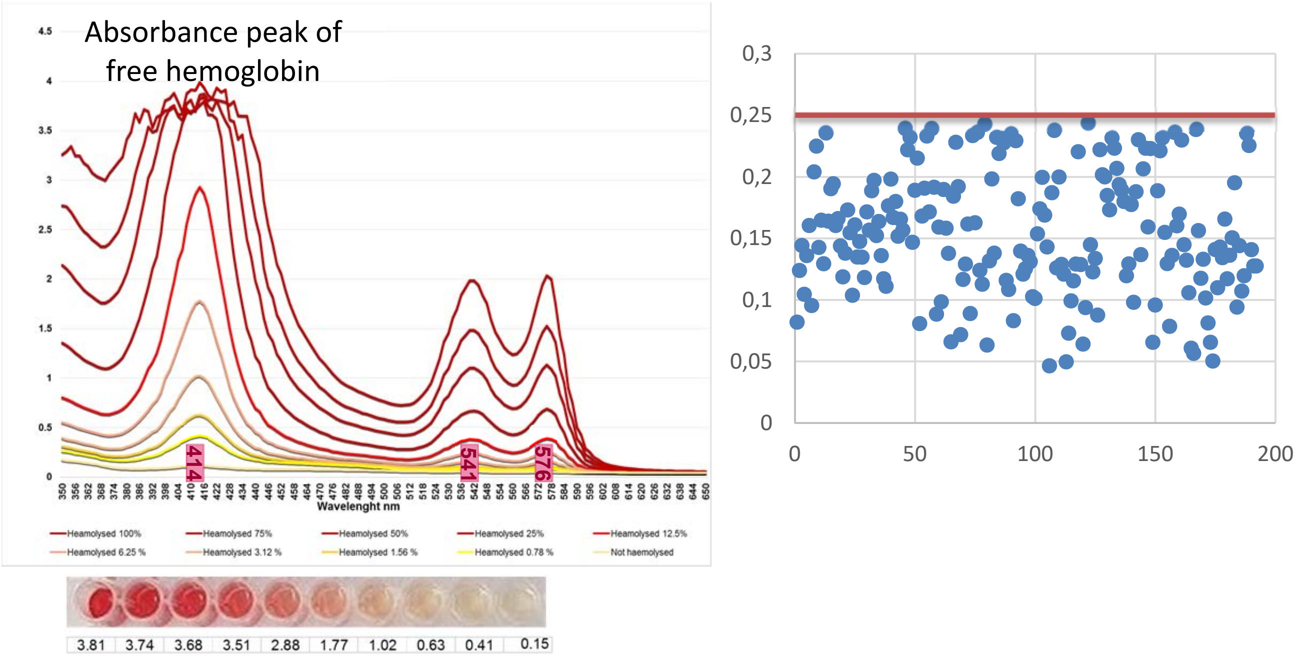

Supplementary Figure S1. Levels of free hemoglobin measured in the plasma samples

Hemolysis was assessed by spectrophotometry at wavelengths from 350 to 650 nm. A dilution series of lysed red blood cells in plasma was prepared (below the chart). The degree of hemolysis was determined based on the optical density (OD) at 414 nm (absorbance peak of free hemoglobin, called Soret band), with additional peaks at 541 and 576 nm. Samples were classified as being hemolysed if the OD at 414 exceeded 0.25. The integrated scatter plot of plasma samples comprises values from 0.04 to 0.25 indicating that the samples were non-hemolysed.

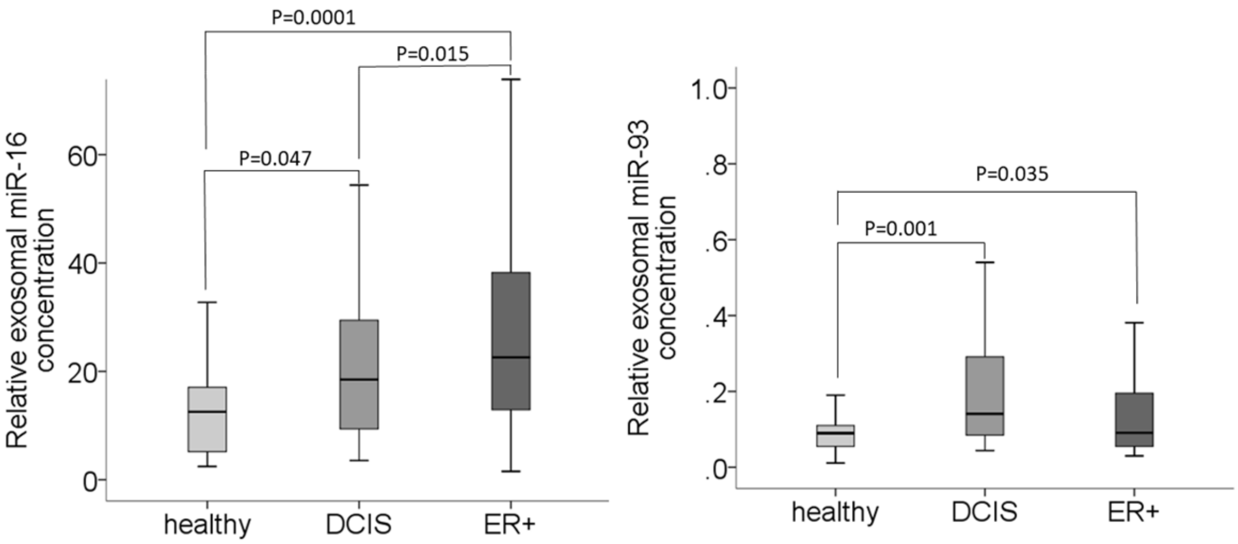

Supplementary Figure S2. Deregulated levels of exosomal miR-16 and miR-93 in DCIS and ER-positive BC patients

Both box blots show the plasma levels of exosomal miR-16 and miR-93 in 39 healthy women, 42 DCIS patients and 81 ER-positive BC patients. P-values are indicated.

Table S1. Summary of the deregulated exosomal miRNAs using array cards containing 48 miRNAs

Populations	No.		miR-15b	miR-16	miR-20a	miR-24	miR-25	miR-30b	miR-30c	miR-93	miR-222	miR-451
All BC vs. Healthy	32 vs. 8	fold change	0.5	2.0	1.1	1.1	1.9	1.2	0.8	**2.7**	1.3	1.9
		p-value	0.576	0.093	1.0	1.0	0.224	1.0	1.0	**0.012**	0.466	0.174
Primary vs. Healthy	16 vs. 8	fold change	1.1	**3.7**	2.5	2.4	2.7	2.6	1.7	**4.3**	1.9	2.4
		p-value	1.0	**0.014**	0.118	0.307	0.081	0.081	0.307	**0.038**	0.116	0.079
Recurrence vs. Healthy	16 vs. 8	fold change	0.3	1.3	0.6	0.6	1.4	0.7	0.5	1.2	1.0	1.6
		p-value	0.171	0.695	0.307	0.433	0.678	0.319	0.080	0.116	1.0	0.373
Primary vs. Recurrence	16 vs. 16	fold change	3.4	**2.8**	**4.4**	4.0	1.9	**4.0**	**3.2**	2.1	1.9	1.5
		p-value	0.099	**0.034**	**0.008**	0.066	0.256	**0.008**	**0.021**	0.318	0.177	0.480
DCIS vs. Healthy	8 vs. 8	fold change	2.5	**9.8**	**6.2**	**6.4**	**4.5**	**3.2**	**2.4**	**21.8**	**3.3**	**6.2**
		p-value	0.267	**0.001**	**0.016**	**0.024**	**0.038**	**0.038**	**0.046**	**0.001**	**0.010**	**0.007**
All BC vs. DCIS	32 vs. 8	fold change	**0.2**	**0.2**	**0.2**	**0.2**	0.4	**0.4**	**0.4**	**0.1**	**0.4**	**0.3**
		p-value	**0.007**	**0.004**	**0.012**	**0.015**	0.557	**0.037**	**0.016**	**0.007**	**0.023**	**0.025**
Primary vs. DCIS	16 vs. 8	fold change	0.5	0.4	0.4	0.4	0.6	0.8	0.7	**0.2**	0.6	0.4
		p-value	0.320	0.065	0.229	0.324	0.613	1.0	0.648	**0.044**	0.273	0.082
Recurrence vs. DCIS	16 vs. 8	fold change	**0.1**	**0.1**	**0.1**	**0.1**	0.3	**0.2**	**0.2**	**0.1**	**0.3**	**0.3**
		p-value	**0.002**	**0.001**	**0.002**	**0.003**	0.082	**0.003**	**0.002**	**0.002**	**0.008**	**0.017**

The table only shows those exosomal miRNAs which were significantly deregulated in a patient group/subgroup.
P values with the corresponding fold changes of exosomal miRNAs in bold.

Supplementary Table S2. Exosomal miRNA data derived from single TaqMan real-time PCR using plasma from healthy women, DCIS patients, primary BC patients and recurrent BC patients

healthy	miR-39	miR-484	miR-16	miR-30b	miR-93
19	25.14	25.14	21.46	27.80	27.80
49	25.49	25.59	24.24	30.98	28.50
70	22.39	28.12	20.73	26.18	28.40
76	23.83	24.39	22.82	27.41	28.13
102	23.05	27.43	22.75	28.31	28.76
103	22.71	23.41	20.26	25.18	27.00
108	20.71	22.36	17.85	23.90	29.29
133	24.28	22.22	18.72	25.50	29.70
134	24.91	26.50	20.67	28.00	29.62
135	22.00	22.42	17.68	23.37	25.87
136	21.54	25.43	19.18	24.77	
137	21.11	21.33	16.23	22.61	24.69
138	20.12	20.21	15.24	21.68	23.34
139	22.56	23.63	21.80	24.07	26.72
140	21.78	23.02	18.56	24.36	
142	22.10	22.41	19.52	24.69	28.78
143	20.79	22.19	20.37	22.65	26.18
145	21.36	22.60	19.50	23.88	25.30
147	22.11	24.47	18.30	23.53	25.84
153	21.51	23.75	20.15	23.66	25.83
154	21.73	23.91	19.20	23.93	26.00
155	21.17	22.46	20.55	29.37	26.13
156	21.76	23.96	19.15	24.23	25.90
158	21.14	22.47	20.50	25.97	26.94
159	20.73	22.14	17.74	22.41	24.41
160	19.72	21.37	16.23	19.65	23.94
161	20.11	22.36	17.55	21.46	23.98
162	20.52	21.40	17.31	21.33	24.47
163	21.56	21.40	17.62	21.99	24.86
164	21.52	21.96	18.10	22.85	24.11
165	20.72	22.51	17.31	21.56	25.61
166	23.91	22.88	20.55	24.53	
167	19.89	22.08	19.82	22.36	25.30
169	22.85	24.68	19.92	23.41	
171	23.19	26.51	21.19	29.40	28.00

172	23.95	25.49	21.73	24.25	27.91
173	22.83	25.08	22.22	24.33	26.50
174	22.37	26.13	22.67	23.36	28.00
178	24.09	22.94	21.10	21.92	27.00

DCIS	miR-39	miR-484	miR-16	miR-30b	miR-93
478	22.98	21.24	19.66	25.30	25.00
479	21.31	19.98	17.77	24.62	22.78
490	24.22	19.69	18.86	25.24	27.36
502	21.20	19.26	16.07	22.39	22.01
503	22.71	20.35	19.71	23.92	23.28
506	24.07	24.17	21.63	27.95	27.86
509	23.01	19.04	17.61	23.66	24.43
521	22.21	21.12	17.49	24.62	25.51
531	22.85	23.02	17.61	26.71	25.92
532	30.53	23.25	24.01	24.66	29.06
538	21.74	19.74	16.80	23.09	24.38
565	22.17	17.88	14.56	21.34	22.15
572	23.16	22.31	17.64	25.28	25.76
576	26.12	20.13	18.51	23.45	24.34
610	24.45	20.42	17.28	21.01	25.29
657	23.53	17.79	15.59	24.18	24.22
658	25.65	23.37	21.73	25.97	26.81
664	25.11	18.18	17.90	21.25	23.16
690	22.38	15.20	15.22	22.09	19.51
712	26.46	21.18	19.70	25.12	26.54
716	24.39	20.20	17.90	22.48	24.19
723	29.58	21.26	25.02	26.38	27.49
742	22.99	19.14	18.37	22.63	22.71
743	26.40	20.71	19.36	25.25	27.19
764	24.51	20.88	18.44	23.36	24.39
795	24.97	20.65	20.85	30.62	27.30
804	21.98	16.86	13.96	20.84	20.31
807	21.74	18.78	16.04	20.93	21.87
826	22.18	17.94	15.12	21.46	21.34
829	29.16	24.23	21.35	28.66	30.61
843	22.46	19.12	16.78	22.19	24.32
846	19.81	18.08	14.21	19.70	21.59
848	24.10	20.58	18.55	24.01	26.56

852	18.11	18.17	14.78	21.39	29.28
863	22.99	18.25	16.33	20.05	22.31
875	20.98	18.17	14.97	21.22	22.61
895	22.40	18.36	15.19	21.10	22.42
906	20.73	18.59	15.26	21.32	23.11
915	23.46	22.89	17.41	24.82	25.43
926	22.37	16.62	14.5	17.54	22.14
971	21.48	21.68	17.00	23.45	25.35
990	20.46	18.17	14.50	22.03	22.11

primary	miR-39	miR-484	miR-16	miR-30b	miR-93
463	21.02	25.60	19.73	27.21	
465	19.93	21.27	17.22	21.65	24.63
466	21.88	21.99	17.85	25.98	22.63
467	20.10	22.58	17.67	22.60	26.72
468	20.10	21.22	16.70	23.64	25.87
470	20.86	22.89	19.17	23.62	
510	30.94	26.24	23.23	31.00	29.05
512	24.75	22.38	22.52	24.70	27.00
513	25.47	23.96	23.42	26.54	31.39
719	21.18	18.54	16.74	28.04	25.09
722	25.17	22.93	21.21	25.82	31.10
849	21.99	17.83	15.41	21.42	22.63
851	21.52	18.73	16.52	23.03	25.48
876	21.49	22.54	18.35	23.55	25.74
877	21.91	24.10	19.78	26.15	27.21
878	23.26	25.58	19.75	24.94	27.88
881	21.38	24.87	18.08	23.35	28.65
882	25.44	26.15	25.17	26.66	27.00
884	25.19	23.23	20.63	26.59	27.00
887	21.12	24.26	19.12	25.62	24.14
890	22.17	24.44	20.12	24.13	27.49
891	21.82	23.96	19.43	22.57	25.49
894	25.21	26.78	21.31	25.40	
898	24.81	25.00	21.51	23.59	26.33
900	23.20	24.51	20.51	24.83	23.92
901	22.36	21.64	16.09	20.48	27.34
902	22.69	24.54	19.35	25.30	27.03
905	21.67	23.01	18.88	25.09	26.03

907	23.33	24.40	20.36	24.65	25.00
908	22.13	21.13	17.94	24.20	25.50
909	23.58	23.85	19.74	26.41	26.30
910	21.93	23.42	18.91	23.38	26.00
911	22.84	22.00	17.88	23.68	25.80
913	22.40	24.57	20.00	25.68	27.42
914	24.29	20.02	16.66	23.10	24.29
917	21.90	22.15	17.95	21.98	26.29
952	22.75	20.25	15.29	20.01	23.57
954	22.69	22.98	18.18	24.64	27.53
955	23.26	19.06	14.83	20.89	22.71
437	21.72	22.64	17.78	22.42	26.60
438	21.26	23.35	16.22	23.33	26.53
443	21.67	23.55	19.82	25.10	
445	22.13	23.26	17.22	22.99	27.80
452	21.22	22.71	16.72	23.16	27.00
474	21.28	24.73	18.50	25.59	28.00
487	21.69	22.91	15.41	21.02	24.49
501	21.95	21.97	15.86	23.11	26.16
563	18.50	16.54	13.24	19.43	20.58
566	22.51	26.17	20.45	23.20	25.90
570	21.12	24.89	18.52	23.34	26.70
607	22.04	25.06	19.55	25.92	26.60
616	25.39	25.67	20.27	25.90	27.15
617	22.05	23.06	17.04	24.38	26.03
624	22.16	21.96	16.97	23.48	26.71
638	21.24	22.27	16.81	21.61	24.93
639	24.10	21.39	19.00	25.31	
648	24.85	24.74	19.96	24.54	26.22
734	20.96	24.77	21.48	26.14	
769	21.68	23.08	18.79	22.93	24.90
803	20.48	26.25	18.14	25.63	26.10
899	25.41	24.31	19.26	27.80	27.17
904	21.76	23.48	17.55	23.31	
916	21.11	24.53	20.14	21.90	25.50
928	20.08	24.17	18.30	23.11	26.69
933	23.60	25.58	19.05	22.77	26.19

recurrence	miR-39	miR-484	miR-16	miR-30b	miR-93
434	22.55	15.89	14.89	22.07	23.82
440	24.43	16.31	14.06	22.14	21.84
446	23.19	22.98	19.08	25.62	26.50
459	19.12	17.06	13.12	20.05	20.62
469	21.15	21.04	16.34	23.25	24.58
473	19.66	16.27	13.10	18.88	21.44
476	20.06	16.16	12.37	19.17	21.28
511	21.26	17.02	15.07	20.98	22.60
533	24.54	18.17	16.19	23.32	23.72
550	20.79	16.33	13.19	18.53	20.87
558	28.26	20.58	19.28	24.10	28.76
578	24.55	19.98	16.92	24.03	25.20
579	24.81	19.54	17.77	22.84	26.87
604	24.80	19.56	17.04	23.40	25.18
609	22.11	17.76	16.07	22.60	24.01
615	23.12	19.76	19.34	24.17	26.14
623	26.14	21.98	19.47	25.33	26.69
625	30.78	23.65	21.84	27.20	30.59
629	24.56	19.27	18.36	24.65	25.65
630	22.57	19.29	16.62	23.39	25.47
649	21.03	19.66	14.54	20.89	24.49
652	22.06	22.14	17.22	25.36	26.04
671	22.88	17.13	12.85	20.71	22.05
677	26.89	19.17	17.43	23.97	25.75
691	24.16	20.02	18.11	23.85	26.39
698	21.32	19.78	19.04	22.75	24.32
710	23.13	19.24	15.24	23.30	29.80
718	23.30	20.72	17.25	22.40	26.04
724	26.36	21.72	20.44	31.00	28.00
726	23.32	21.33	21.19	23.95	26.16
741	22.91	21.02	19.21	30.02	26.20
746	20.29	20.79	14.63	23.49	24.98
749	22.45	22.95	17.13	25.37	26.61
752	20.34	19.10	16.55	22.93	24.31
754	28.67	22.34	20.98	23.92	29.77
772	22.88	20.63	15.91	24.47	25.71
778	23.54	22.32	20.94	26.36	26.84
799	20.46	19.92	14.11	21.96	24.23
800	20.09	19.25	16.59	23.02	23.79
812	22.05	20.94	17.83	24.21	26.69

827	25.45	22.45	18.47	25.64	27.28
839	21.77	20.93	15.52	21.27	22.74
850	22.81	19.54	17.20	23.50	26.11
855	23.34	22.37	20.91	25.02	25.20
870	19.14	17.75	14.25	20.17	22.35
993	19.34	17.80	14.18	20.56	23.25
